# FunClust: a web server for the identification of structural motifs in a set of non-homologous protein structures

**DOI:** 10.1186/1471-2105-9-S2-S2

**Published:** 2008-03-26

**Authors:** Gabriele Ausiello, Pier Federico Gherardini, Paolo Marcatili, Anna Tramontano, Allegra Via, Manuela Helmer-Citterich

**Affiliations:** 1 Centre for Molecular Bioinformatics, Department of Biology, University of Rome “Tor Vergata”, Rome, Italy; 2 Department of Biochemical Sciences, University of Rome “La Sapienza”, Rome, Italy

## Abstract

**Background:**

The occurrence of very similar structural motifs brought about by different parts of non homologous proteins is often indicative of a common function. Indeed, relatively small local structures can mediate binding to a common partner, be it a protein, a nucleic acid, a cofactor or a substrate. While it is relatively easy to identify short amino acid or nucleotide sequence motifs in a given set of proteins or genes, and many methods do exist for this purpose, much more challenging is the identification of common local substructures, especially if they are formed by non consecutive residues in the sequence.

**Results:**

Here we describe a publicly available tool, able to identify common structural motifs shared by different non homologous proteins in an unsupervised mode. The motifs can be as short as three residues and need not to be contiguous or even present in the same order in the sequence. Users can submit a set of protein structures deemed or not to share a common function (e.g. they bind similar ligands, or share a common epitope). The server finds and lists structural motifs composed of three or more spatially well conserved residues shared by at least three of the submitted structures. The method uses a local structural comparison algorithm to identify subsets of similar amino acids between each pair of input protein chains and a clustering procedure to group similarities shared among different structure pairs.

**Conclusions:**

FunClust is fast, completely sequence independent, and does not need an *a priori* knowledge of the motif to be found. The output consists of a list of aligned structural matches displayed in both tabular and graphical form. We show here examples of its usefulness by searching for the largest common structural motifs in test sets of non homologous proteins and showing that the identified motifs correspond to a known common functional feature.

## Background

More than a hundred methods have been developed so far for the automated discovery of unknown short conserved motifs in a set of protein or nucleic acid sequences [1]. These methods are routinely used for the identification of functional features, such as, for example, transcription factor binding sites in a set of gene regulatory regions.

However a functional motif needs not to be contiguous in sequence and might arise from the clustering in space of similar side chains coming from different parts of non homologous proteins. Finding occurrences of shared structural motifs can be instrumental for mapping the interaction site of different proteins with the same partner [2], for locating of the binding site for a common ligand even of unknown identity or for identifying an epitope shared, for example by an external agent and an endogenous protein involved in autoimmune diseases.

At present, several applications for the comparison of multiple structures are available [3,4] and are used for clustering protein structures in families or for identifying large structural motifs shared by different folds. All these algorithms are based on fold comparison methods, and therefore the alignments they produce are often sequence-dependent and/or require at least a small core of conserved residues which are contiguous in the primary sequence [5,6].

These fold comparison methods cannot be used to identify small structural motifs not conserved in sequence and belonging to non homologous proteins. Nevertheless identifying such motifs is important to study cases where the same metabolite, for example ATP, is bound to proteins with different folds [7,8], or where the same protein interacts with different partners using the same surface [9].

Here we describe FunClust [10], a new web server for the identification of common structural motifs in a set of non homologous protein structures without any knowledge about the type or position of the motif, which, additionally, does not need to be present in all the submitted structures.

FunClust is based on a local (as opposed to global) structural comparison program [11]. Local structural comparison methods [12-15] can identify small sets of residues organized in a conserved geometry, irrespectively of the order in which they appear in the primary sequence. They are useful to search for functional and/or structural motifs such as active sites or ligand binding sites in non-homologous protein structures [16].

In this work we show that our method is able to effectively and efficiently identify common functional and/or structural motifs present in different structures. As test cases, we used proteins known to share common motifs and a complete set of ATP binding proteins. The server is also being used for finding common interface patches in proteins interacting with the same partner [2].

## Results

### The FunClust web server

The FunClust web server [10] enables the quick identification of structural motifs, putatively associated with a common function, present in an ensemble of non-homologous protein structures. Users submit a set of protein structures deemed to share a given function, binding capability or a common epitope, without the need to specify where the common structural motif should be located. The output consists of a list of one or more conserved sets of residues.

### Input

The method accepts as input a set of PDB [17] codes or user-submitted coordinates in PDB file format. In the latter case, it is also possible to provide an arbitrary subset of residues instead of the whole protein. If a PDB code is given without any chain identifier, all the available chains are considered in the computation.

PDB codes and user-submitted coordinates are processed in order to validate their format and detect sequence similarities that can indicate homology relationships. Only one representative structure is used for each set of submitted protein chains with sequence identity higher than a user-specified threshold. This restriction reduces or eliminates the risk of finding local similarities arising because of the overall similarity among the input proteins.

The user can set the value of five parameters: the maximum r.m.s.d. among the involved residues, the maximum distance between the side chains of the residues forming the motif, the inclusion/exclusion of solvent accessible residues only, the inclusion/exclusion of hydrophobic residues and the weight of physicochemical similarities among the matched residues.

A set of pre-selected combinations of parameters tailored to the identification of four different kinds of functional sites (active sites, ligand binding sites, protein-protein interfaces and hydrophobic core packing motifs) is also provided.

### Output

**Figure 1:**
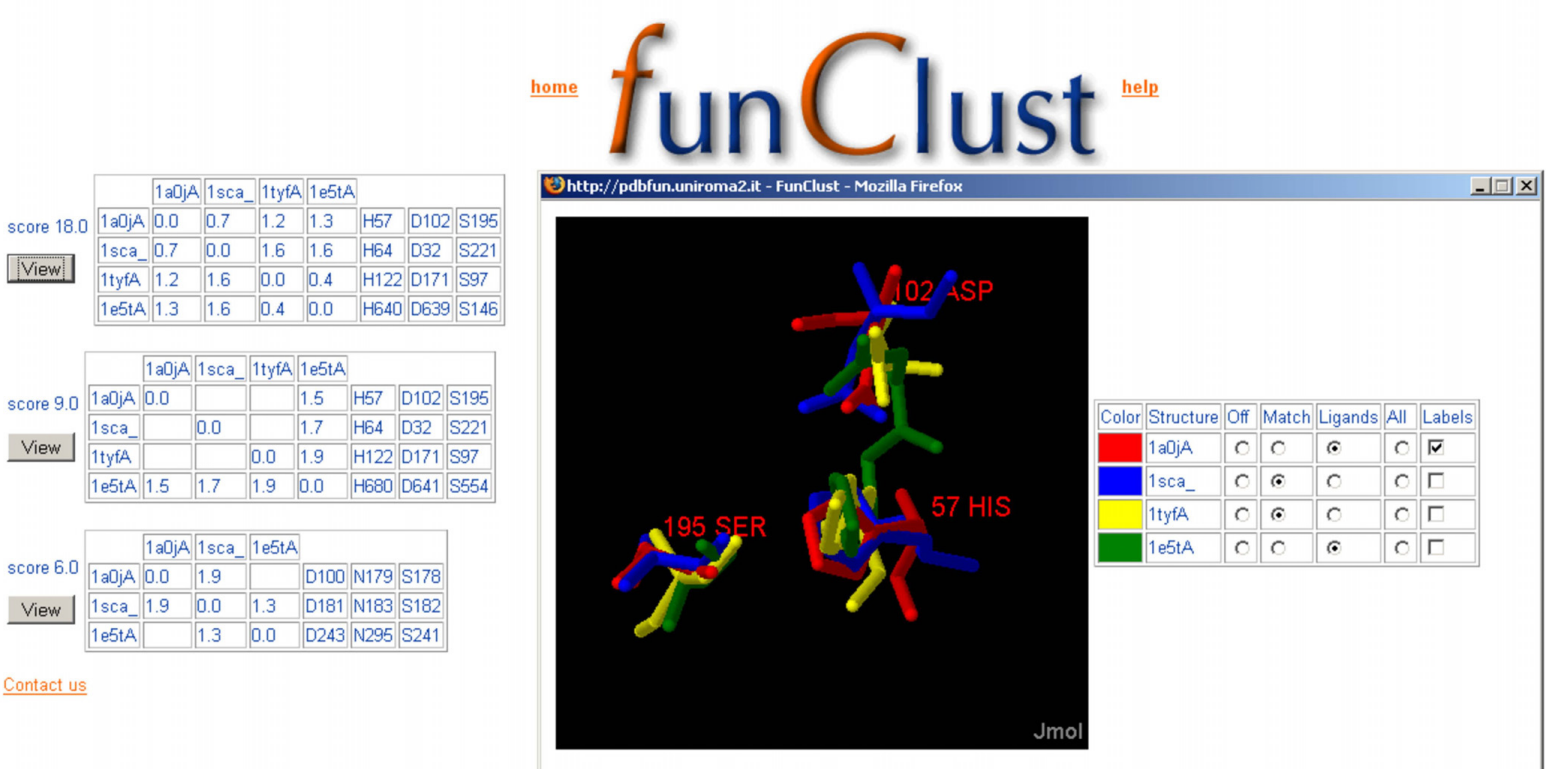
**Details of structural matches.** Output page of a FunClust search in four non-homologous serine protease protein chains (1a0jA, 1sca, 1tyfA and 1e5tA). The three different clusters identified are shown in tabular form. For each cluster the associated score is reported. The right section of each table reports, in each row, the residues belonging to the different structures, with structurally aligned residues written in the same column. The left section shows the r.m.s.d. value for each match identified between the structures corresponding to the row and column of the table (recall that two structures belonging to the cluster do not necessarily match to each other). In the example shown, the first of the three clusters is composed of the four catalytic triads which are therefore correctly identified. The second cluster identifies three non-catalytic residues in structure 1e5t, while the third one (with the lowest score) involves only three of the four structures. A user activated popup window shows a graphical view (created using the Jmol applet) of the first cluster. The four different structures have been superposed on the residues belonging to the structural motif. Each structure has a different colour, and only the residues involved in the cluster are shown. Commands to trigger the display of the whole structure and of the labels for each protein in the cluster are located in the right portion of the window.

The output is a list of all the identified structural motifs, sorted by an approximate significance score, as described below. Each motif consists of a set of at least three protein chains and at least three residues in each chain. Not all the user-submitted structures need to contain the motif, but all the identified structures must contain a motif formed by the same number of residues. Each motif is shown in the output page along with its score in tabular and graphical form (figure 1). In the tabular form, the residues of each structure are listed in a different row, with the corresponding residues in different structures aligned in the same columns.

Each motif is associated to its approximate significance score, which depends upon the number of protein chains containing it. More specifically, the score is given by the number of residues in the motif multiplied by the number of aligned pairs of structures. The score of a cluster formed by 3 aligned residues in 5 proteins chains can range from 3*10 (the maximum number of pairs formed by 5 structures) to 3*4 (the minimum number of pairs compatible with a motif identified in five structures).

### Test cases

We show here some examples where the largest identified structural cluster of residues shared by a set of protein structures corresponds to a known functional motif. The examples are taken using PROSITE [18] regular expressions, CSA [19] catalytic residues, pdbFun residues in binding sites [20] and ELM [21] motifs. We selected our cases from the limited number of functional motifs identified on at least three structures with a completely different fold and low sequence identity. For each selected motif, we requested the representing structures to have a different CATH [22] architecture and share a sequence identity lower than 25%.

Some examples of cases where the first structural cluster identified by the server correctly pairs some or all of the correspondent functional residues in all the submitted structures are the serine endopeptidases enzymes (from CSA), the EF HAND motif (from ELM), the zinc binding site (from pdbFun) and the 4Fe-4S ferredoxin pattern (from PROSITE).

In Table 1 we show the results of the FunClust server for each different functional motifs. We also report the score and the set of parameters used (r.m.s.d. and side chain proximity).

**Table 1:** Test cases

Function	Source	Score	Proximity	Rmsd	PDB	Chain	CATH	Matched Residues
Serine endopeptidases EC 3.4.21	CSA	18	H	H	1a0j	A	2.40.10.10	H57 D102 S195
					1sca		3.40.50.200	H64 D32 S221
					1tyf	A	3.90.226.10	H122 D171 S97
					1e5t	A	3.40.50.1820	H640 D639 S146

WW domain	PROSITE	9	H	L	1eg3	A	NA	W61 N75 T78
					1o6w	A	2.20.70.10	W4 N18 T21
					1zcn	A	NA	W11 N26 T29

4Fe-4S ferredoxin	PROSITE	42	L	L	1a6l		3.30.70.20	C16 C45 C49 C20 P50 P21 C42
					1jb0	C	1.20.1130.10	C53 C16 C20 C57 P21 P58 C13
					1kf6	B	3.10.20.30	C210 C154 C158 C214 P159 P215 C151

EF HAND	ELM	9	M	H	1bmo	A	1.20.238.10	D257 D259 N260
					1daq	A	3.30.60.30	D40 D44 N42
					1aj5	A	1.10.1330.10	D227 D229 N230

LIMDomain	PROSITE	12	H	L	1a7i		2.10.110.10	C10 C13 H31 C34
					1wig	A	NA	C34 C37 H56 C59
					2cuq	A	NA	C18 C21 H38 C41

Zn binding	PDBFUN	45	H	M	1a5t		3.40.50.300	C62 C65 C50
					1a73	A	3.90.75.10	C125 C132 C138
					1adn		3.40.10.10	C72 C69 C38
					1adt		NA	C450 C467 C398
					1ajy	A	NA	C50 C60 C34
					1b55	A	2.30.29.30	C155 C154 C165

In this table six cases of functional motifs are reported that the server identified as the largest cluster of conserved residues. The score, r.m.s.d. and side chains maximum proximity parameters are reported (H is high, M is medium and L is low). A detailed parameter description can be found in the online help.

For each motif the list of submitted PDB structures is present along with their CATH code. Finally, for each structure the aminoacids that have been included in the common cluster are indicated.

### ATP binding pockets

As an additional test of our method, we evaluated its ability to identify a common structural motif in a set of protein structures binding the same ligand. We used a complete set of 57 ATP binding structures [23] sharing less than 35% sequence identity, representative of all the PDB. For the comparison, all residues having at least one atom at less than 4.5Å from the bound ATP were used. Using the standard ligand binding site parameters of FunClust, a total of four structural motifs (cluster of residues) were identified. The three highest scoring clusters (96, 90 and 90) are composed respectively of 13, 16 and 19 different proteins all sharing a set of three residues: two Glycines and a Lysine or Serine. All residues in the three identified motifs belong to the p-loop binding motif [24]. By superposing the motifs, it can be seen that the vast majority of ATP molecules appear to have one of their phosphate atoms in the same location. The complete set of results can be viewed on the server web pages, using the “Example 3” set of structures.

## Conclusions

Here we present a new server for the multiple local alignment of protein structures and show some examples of its application to the discovery of common functional patterns in serine endopeptidases, EF HAND containing proteins, ferredoxins, zinc and ATP binding proteins. FunClust is a useful tool in the automated discovery of local structural motifs shared by a set of non-homologous protein structures. The server is fast and easy to use. To date, this is the only method available on the web for the automated and unsupervised identification of local structural motifs in unrelated protein structures.

## Methods

FunClust uses two different algorithms: a local structural comparison method that is able to identify all the similarities between a single pair of structures, and a procedure that searches for clusters of matches involving residues common to different structures. The result of the two procedures is a list of local structural motifs, each one identified by a cluster of structural matches between different pairs of structures.

### Local comparison algorithm

In the first step of the procedure, all the input chains are compared pairwise using Query3D [11] a fast local comparison algorithm. This step involves, for n protein chains, n*(n-1)/2 comparisons. The method is able to find all the subsets of at least three residues that can be superposed within a given r.m.s.d. value and with sequence similarity above a user-defined threshold. The r.m.s.d is calculated using a two-point representation of each residue, comprising the C-alpha and the side-chain geometric centre.

Each comparison run generates a list of one or more sequence-independent local structural matches between the two proteins.

### Clustering of structural matches

In the second step of the procedure a clustering algorithm identifies the largest structural matches shared by the highest number of structures.

To this end, a graph is built in which every node represents a match. Edges are drawn between nodes representing matches belonging to different lists (i. e. different pairs of structures involved in the comparison), according to the following criteria:

1. the two lists must have one structure in common, i. e. they represent matches of the same structure with two different targets;

2. the matches to be connected share at least three residues.

This graph is analyzed by a fast and simple procedure that searches for the largest number of connected nodes containing no more than one match from every list and at least three common residues in all the involved structures.

The algorithm selects the highest scoring set of connected matches in the graph. The score is given by the number of residues in common between all the matches multiplied by the number of matches belonging to the cluster.

When the highest scoring cluster is identified, the corresponding matches are removed from the graph and the search is repeated until all clusters are identified. Each cluster of matches corresponds to a different structural motif, with the score of the motif being that of the cluster.

### Implementation notes

Both the comparison and clustering of multiple protein structures are complex problems, but extensive testing demonstrated that the CPU cost of our algorithm is fully compatible with protein structures of reasonable size and with motifs of average size present in up to 20 different structures. Comparison times range from fractions of a second to a few minutes. However, a time limit of 1 minute is given to web server users. Web pages have been tested using the most common browsers for Windows, Mac and Linux platforms.

## Competing interests

The authors declare that they have no competing interests.

## Authors' contributions

GA conceived the study, carried out the work and drafted the manuscript. PM helped in testing the server. PFG, AV and AT participated in the design of the work. MHC participated in the design of the work and in its coordination. All authors read and approved the final manuscript.
